# A case of B-cell acute lymphoblastic leukemia in a child with Down syndrome bearing a t(2;12)(p12;p13) involving *ETV6* and biallelic *IGH@* rearrangements

**DOI:** 10.1186/s40364-015-0036-1

**Published:** 2015-06-05

**Authors:** Carlos A. Tirado, David Shabsovich, Yeun Kim, Peter Traum, Sheeja Pullarkat, Michael Kallen, Nagesh Rao

**Affiliations:** Department of Pathology and Laboratory Medicine, David Geffen School of Medicine at UCLA, Los Angeles, CA USA

**Keywords:** *ETV6*, B-ALL, Cytogenetics, FISH

## Abstract

**Background:**

Rearrangements involving *ETV6* (12p13) are among the most common structural abnormalities in pediatric B-cell acute lymphoblastic leukemia (B-ALL) and involve numerous partner genes. Additionally, the t(8;14)(q11.2;q32), which can result in the placement of *CEBPD* (8q11.2) near the regulatory regions of *IGH@* (14q32) and consequent overexpression of *CEPBD*, occurs at a higher frequency in individuals with Down syndrome-associated ALL (DS-ALL) compared to both the general and pediatric population. The coexistence of cytogenetically detectable *ETV6* abnormalities and t(8;14)(q11.2;q32) is a rare occurrence in B-ALL and has only been reported in a single case in the literature.

**Findings:**

Herein, we present a case of B-ALL in a 9-year old male with Down syndrome in which conventional cytogenetic analysis revealed two reciprocal translocations: a t(8;14)(q11.2;q32) and a t(2;12)(p12;p13). Interphase and metaphase fluorescence in situ hybridization (FISH) analysis using break apart probes confirmed the involvement of *IGH@* and *ETV6* in these translocations, respectively. Additionally, interphase FISH revealed a clonal subpopulation bearing biallelic *IGH@* rearrangements not observed by conventional cytogenetic analysis.

**Conclusions:**

To the best of our knowledge, this is the first reported case of B-ALL bearing an *ETV6* translocation with a partner gene on the short arm of chromosome 2 confirmed by FISH. Additionally, it is the second reported case of t(8;14)(q11.2;q32)-ALL bearing a concomitant, cytogenetically detectable abnormality involving *ETV6*. This case provides insight into a novel translocation involving *ETV6* as well as potentially unique and understudied mechanisms of clonal evolution in pediatric B-ALL.

## Background

Pediatric B-acute lymphoblastic leukemia (B-ALL) often bears a range of numerical and structural cytogenetic abnormalities, including but not limited to the following, in decreasing frequency: t(12;21)(p13;q22) [*ETV6/RUNX1*], hyperdiploidy, t(1;19)(q23;p13.3) [*PBX1/TCF3*], t(4;11)(q21;q23) [*AFF1/MLL*], hypodiploidy, and t(9;22)(q34;q11.2) [*BCR/ABL1*] [1]. Additionally, the t(8;14)(q11.2;q32), which can result in the placement of *CEBPD* (8q11.2) near the regulatory regions of *IGH@* (14q32) and consequent overexpression of *CEPBD*, occurs at a much higher frequency in individuals with Down syndrome compared to both the general and pediatric population and is associated with an intermediate prognosis [2].

Rearrangements involving *ETV6* (12p13) are among the most common structural abnormalities in pediatric B-ALL. The t(12;21)(p13;q22), the most common of these translocations, results in the production of a chimeric transcription factor bearing the DNA-binding domain of *RUNX1* (21q22) and the transactivation domain of *ETV6* (12p13), resulting in aberrant activation of genes regulated by *RUNX1*. Although the t(12;21) [*ETV6/RUNX1*] is the most common of these rearrangements, other translocations involving *ETV6* with greater than 20 partner genes have been observed, including protein tyrosine kinases and transcription factors, many of which can act by distinct mechanisms to promote leukemogenesis [3]. Additionally, other anomalies involving *ETV6* have been observed in various hematological malignancies ranging from deletions, to point mutations, to alterations at the epigenetic level, to amplifications [3–5]. Although abnormalities involving *ETV6* are a relatively common finding in B-ALL, the precise leukemogenic role of the gene in the context of some of the aforementioned aberrations remains understudied.

## Case presentation

A 9-year old male with Down syndrome presented with persistent fever and fatigue. Complete blood count revealed pancytopenia (WBC 3.01×10^3^/μL, RBC 2.87×10^6^/μL, platelet count 43×10^3^/μL) with a differential of 32 % lymphoblasts, 52 % lymphocytes, 7 % neutrophils, 4 % monocytes, 1 % metamyelocyte, and 1 % myelocyte. Flow cytometry on peripheral blood revealed excess abnormal blasts comprising 22 % of total cells, and expressing CD34, CD10, CD19, CD22, and HLA-DR. A bone marrow biopsy showed hypercellular marrow (~90 %) and 95 % replacement by sheets of lymphoblasts. These findings are consistent with a diagnosis of B-lymphoblastic leukemia, and thus, a diagnosis of B-ALL was rendered. Induction chemotherapy was immediately started with Vincristine and cytarabine. On day 29 post induction chemotherapy, a bone marrow biopsy showed variably lower cellular marrow with approximate overall cellularity of 80 %. A follow-up bone marrow biopsy showed minimal residual disease, displaying a favorable response to therapy.

## Methods

### Conventional cytogenetics

Chromosome analysis was performed on 30 metaphase spreads from bone marrow and peripheral blood using standard cytogenetic techniques. Karyotypes were prepared using Applied Imaging CytoVision software (Applied Imaging, Genetix, Santa Clara, CA) and described according to the International System for Human Cytogenetic Nomenclature (ISCN) 2013 [6].

### Fluorescence in situ hybridization (FISH)

FISH was performed on interphase nuclei and/or previously G-banded metaphase spreads using the following probes acquired from Abbott Molecular (Abbott Molecular, Des Plaines, Illinois 60018):

Vysis LSI ETV6/RUNX1 ES Dual Color Translocation Probe Set

Vysis LSI ETV6 Dual Color, Break Apart Probe Kit

Vysis LSI IGH Dual Color, Break Apart Rearrangement Probe

Vysis LSI BCR/ABL + 9q34 Tricolor, Dual Fusion Translocation Probe

Vysis LSI MLL Dual Color, Break Apart Rearrangement Probe

Vysis LSI PDGFRB (Cen) FISH Probe

Vysis LSI PDGFRB (Tel) SpectrumGreen FISH Probe

Vysis CEP4 Probe

Vysis CEP10 Probe

## Findings

### Conventional cytogenetics

**Fig. 1:**
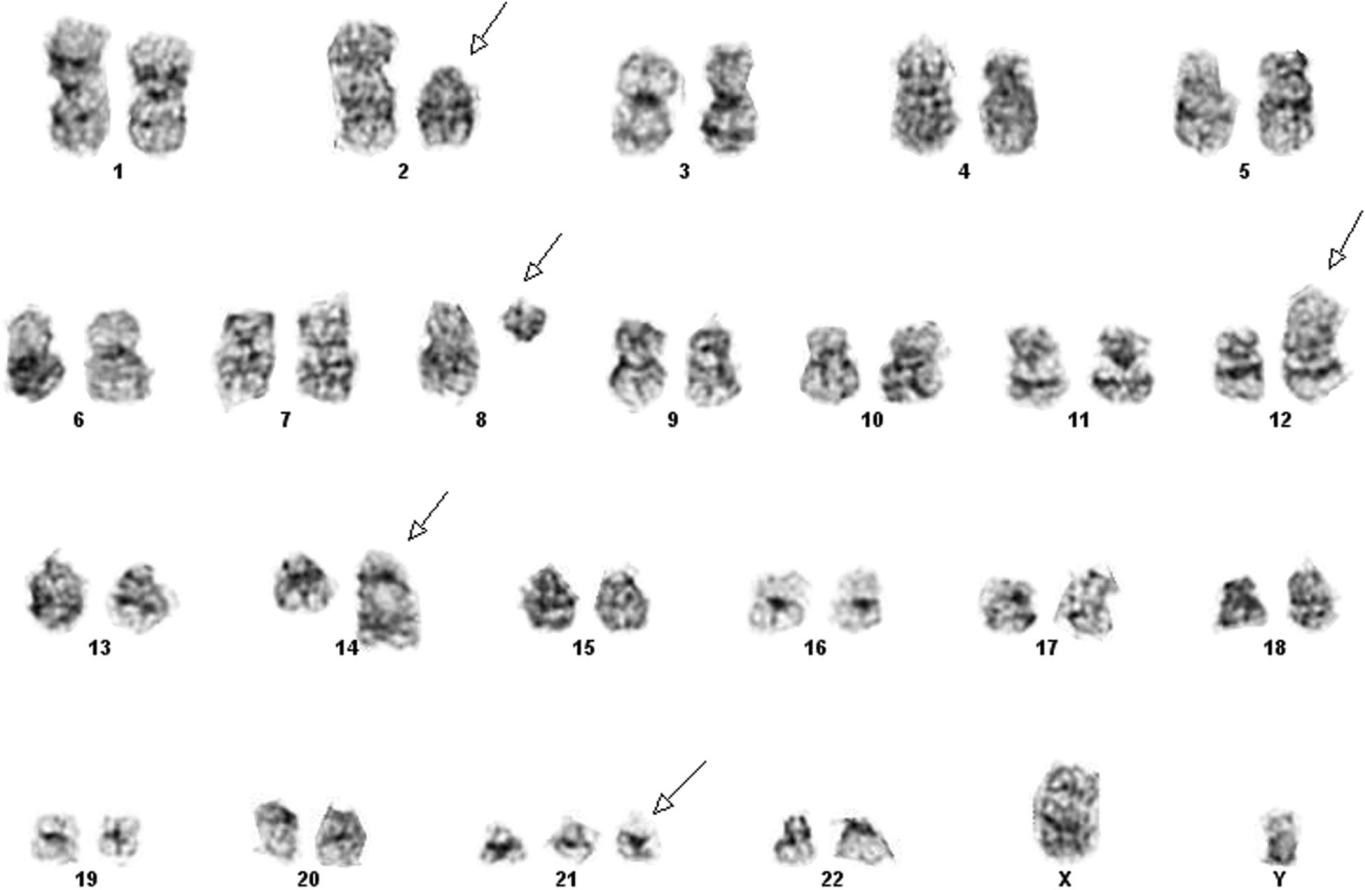
Abnormal karyotype from metaphase spread seen on G-banded chromosomes in the patient’s bone marrow: 47,XY,+21c[25]/47,idem, t(2;12)(p12;p13),t(8;14)(q11.2;q32)[4]

Chromosome analysis of the bone marrow revealed 5 out of 30 metaphase spreads with two reciprocal translocations involving 2p12 and 12p13 as well as 8q11.2 and 14q32 (Fig. 1). All 30 cells examined exhibited trisomy 21 (+21). No cytogenetically normal cells were observed.

The karyotype of the bone marrow of this patient was described as: 47,XY,+21c[25]/47,idem,t(2;12)(p12;p13), t(8;14)(q11.2;q32)[5].

### Fluorescence in situ hybridization (FISH)

**Fig. 2:**
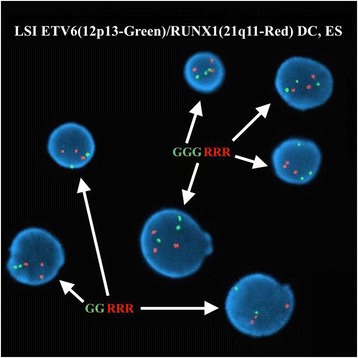
FISH studies on interphase nuclei using Vysis LSI ETV6/RUNX1 Dual Color, Extra Signal Probe reveal 3 signals for *ETV6* in 50.7 % (152/300) of the nuclei examined

**Fig. 3:**
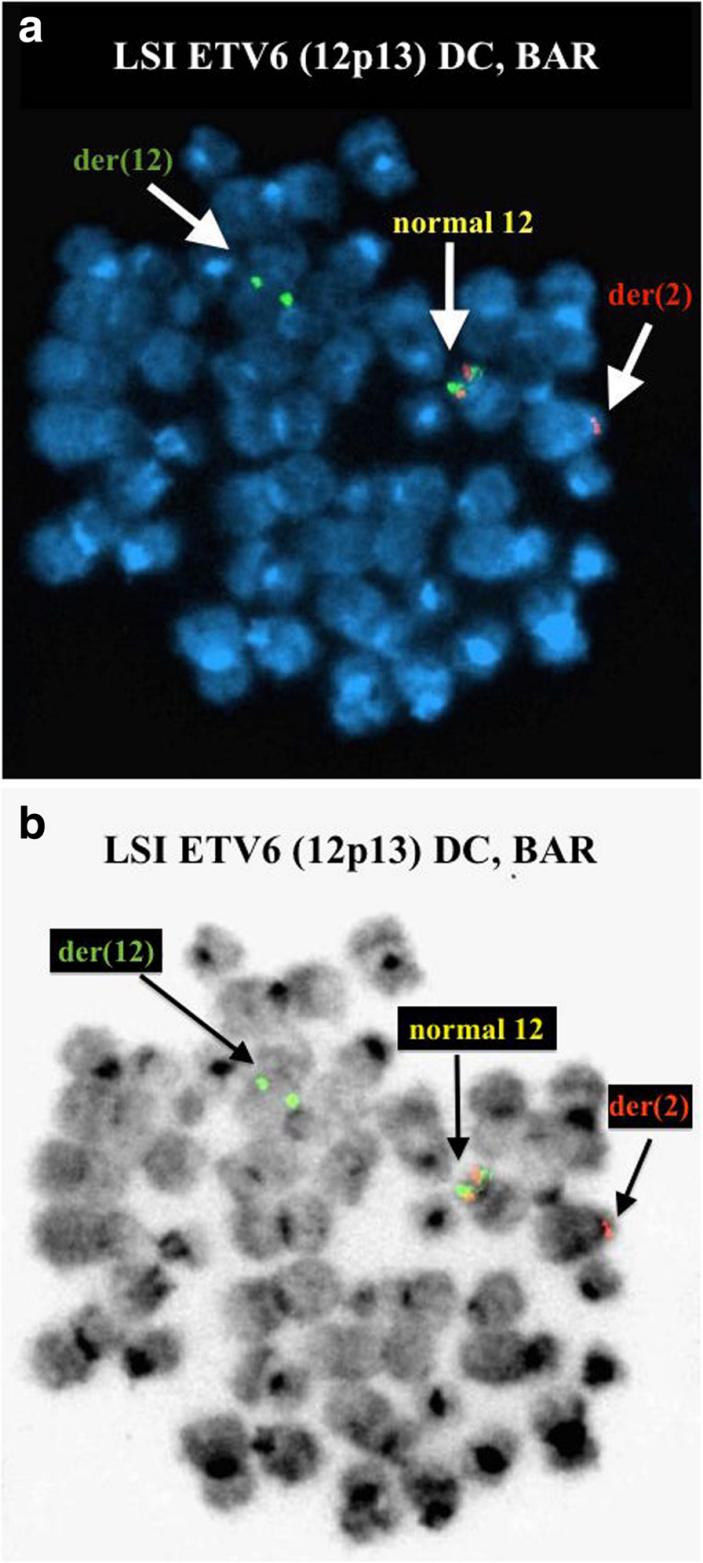
FISH on previously G-banded metaphase spreads using Vysis LSI ETV6 Dual Color, Break Apart Probe Kit reveal a monoallelic split signal. **a** DAPI image. **b** Inverted DAPI image

FISH analysis on interphase nuclei using the Vysis LSI ETV6/RUNX1 ES Dual Color Translocation Probe Set revealed 3 signals corresponding to *ETV6* in 50.7 % (152/300) of the nuclei examined, suggesting a potential *ETV6* (12p13) gene rearrangement (Fig. 2). All 300 nuclei examined exhibited 3 signals corresponding to *RUNX1* (21q22), consistent with the constitutional genetic makeup of this patient (Down syndrome). FISH on previously G-banded metaphase spreads using the Vysis LSI ETV6 Dual Color, Break Apart Probe Kit revealed a fusion signal (F) corresponding to an intact *ETV6* gene on the normal chromosome 12 and a split signal (1R1G) corresponding to a rearranged *ETV6* gene, with a red signal (5′/telomeric region of *ETV6*) on the derivative chromosome 2 and a green signal (3′/centromeric region of *ETV6*) on the derivative chromosome 12, ultimately confirming the t(2;12) observed by conventional cytogenetic analysis (Fig. 3).

**Fig. 4:**
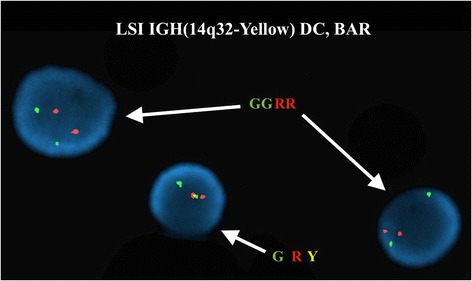
FISH studies using Vysis LSI IGH Dual Color, Break Apart Rearrangement Probe on interphase nuclei showed a monoallelic split signal in 89.6 % (269/300) of the nuclei examined and biallelic split signals in 6.7 % (20/300) of the nuclei examined

**Fig. 5:**
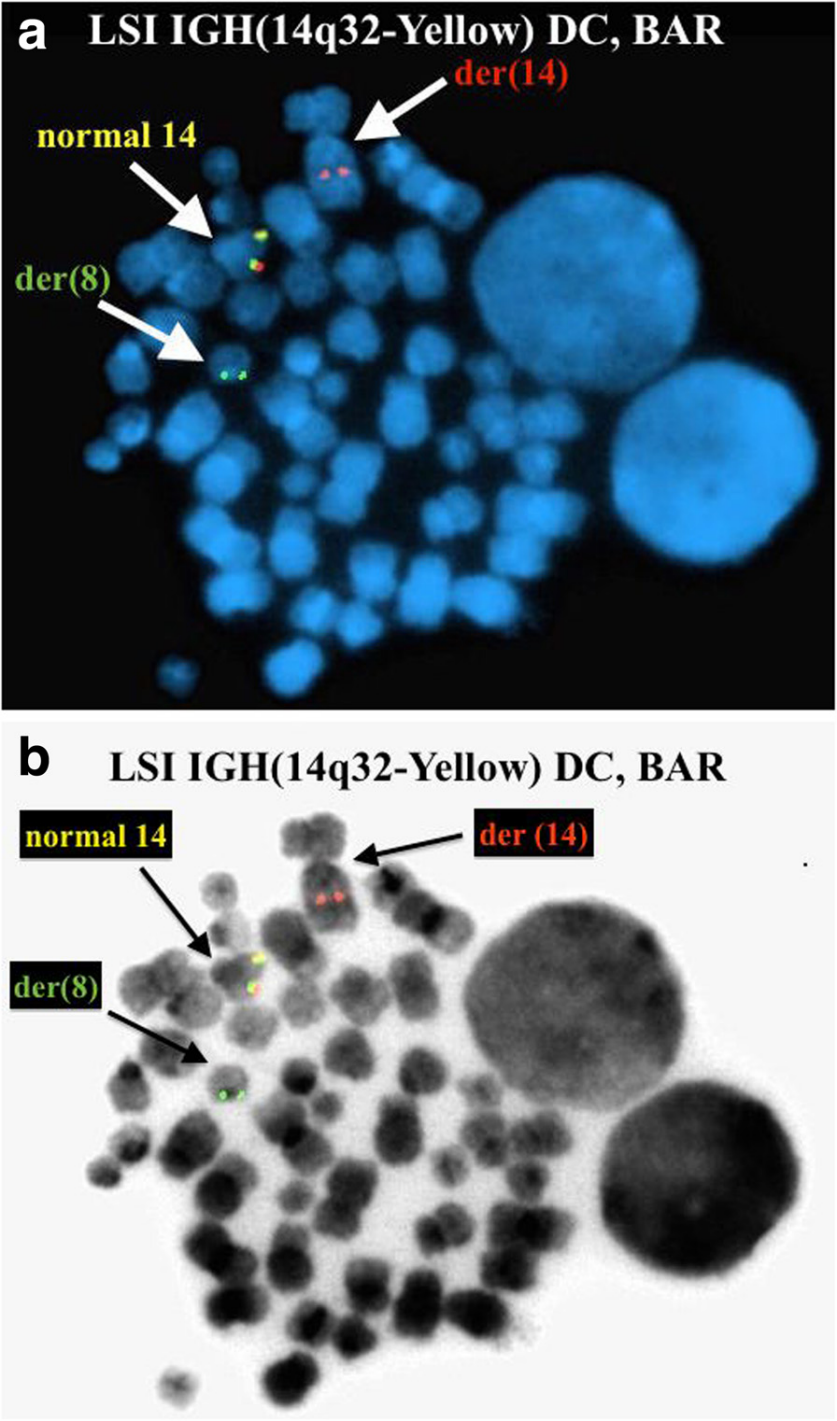
FISH on a previously G-banded metaphase spread using Vysis LSI IGH Dual Color, Break Apart Rearrangement Probe revealed a monoallelic split signal. **a** DAPI image. **b** Inverted DAPI image

FISH analysis on interphase nuclei using the Vysis LSI IGH Dual Color, Break Apart Rearrangement Probe revealed two abnormal clonal populations (Fig. 4). The first clone showed one fusion signal and one split signal (1F1R1G) and was observed in 269/300 (89.7 %) of the nuclei examined. The second clone showed two split signals (2R2G) and was observed in 20/300 (6.7 %) of the nuclei examined. The former suggests a rearrangement involving one *IGH@* gene with the other remaining intact (monoallelic), and the latter suggests rearrangements of both *IGH@* genes (biallelic). FISH on previously G-banded metaphase spreads also revealed the former signal pattern (1F1R1G) consisting of a fusion signal and a split signal (1F1R1G), with the fusion signal corresponding to an intact *IGH@* gene on the normal chromosome 14, the red signal (3′/telomeric region of the *IGH@* gene) on the derivative chromosome 8, and the green signal (5′/centromeric region of the *IGH@* gene) on the derivative chromosome 14, ultimately confirming the t(8;14) observed by conventional cytogenetic analysis (Fig. 5). No metaphase spreads bearing a second 14q32 rearrangement were observed by conventional cytogenetic analysis, and we were thus not able to detect or localize the latter signal pattern (2R2G; two split signals) by metaphase FISH.

Analysis using other probes did not reveal any additional abnormal signal patterns. This constellation of results was described as follows:

nuc ish(ETV6, RUNX1)x3[152/300]/(ETV6x2, RUNX1x3)[148/300].

nuc ish(IGH@*x*2)(3′IGH@ sep 5′IGH@x1)[269/300]/(IGH@*x*2)(3′IGH@ sep 5’IGH@*x*2)[20/300]

nuc ish(ASS1, ABL1, BCR)*x*2[300]

nuc ish(MLLx2)[300]

nuc ish(PDGFRBx2)[300]

nuc ish 4cen(CEP4x2)[300]

nuc ish10cen(CEP10x2)[300]

## Conclusion

The cytogenetic findings in this case highlight a unique combination of rearrangements that has not been previously described in B-ALL. The t(8;14)(q11.2;q32) is a recurrent translocation in B-ALL that generally causes exchange of the regulatory regions of the *IGH@* gene and the *CEBPD* gene, placing *CEBPD* in close proximity to the regulatory regions of *IGH@* and resulting in its overexpression [7]. Although it is relatively rare in the general population, this translocation is more frequent in Down syndrome-associated ALL (DS-ALL) and is associated with a B-cell precursor immunophenotype and an intermediate prognosis [2, 8, 9]. It has also been shown to be associated with a number of secondary cytogenetic abnormalities, including der(14)t(8;14)(q11.2;q32), t(9;22)(q11.2;q34), +21, +X, and +14, although patients with Down syndrome and t(8;14)(q11.2;q32)-positive ALL generally do not bear a concomitant Philadelphia chromosome [2].

The t(2;12)(p12; p13) is a recurrent, albeit rare, translocation in cyclin D1-negative mantle cell lymphoma (MCL). In these cases, it involves the *IGK* (2p12) and *CCND2* (12p13) genes, and results in overexpression of *CCND2* due to the placement of the *CCND2* gene near the regulatory regions of the *IGK* gene [10]. However, in our case, FISH analysis using both a break apart probe and a dual color translocation probe specific to the *ETV6* gene, which is also located at the 12p13 band, confirmed that the t(2;12)(p12;p13) we observed involves the *ETV6* gene, and not *CCND2*. Additionally, since both *ETV6* and *CCND2* localize to 12p13, we confirmed that the *ETV6* break apart probe (Vysis LSI ETV6 Dual Color, Break Apart Probe Kit) we utilized in our analysis bears no overlap with the *CCND2* gene, minimizing the possibility of a false-positive result. The break apart probe we utilized spans chr12: 11321260–12578058, whereas the *CCND2* gene spans chr12: 4382902–4414522. We queried the Mitelman Database of Chromosome Aberrations and Gene Fusions in Cancer for cases of B-ALL bearing a t(2;12)(p11–p13; p12–p13) and were only able to identify seven cases in addition to our case, indicating that it is an extremely rare cytogenetic abnormality in B-ALL (Table 1). None of these cases included corresponding molecular analysis of *ETV6*, so the involvement of the gene in the reported cases is not known. Thus, to the best of our knowledge, this is the first reported case of B-ALL bearing a t(2;12)(p12;p13) with *ETV6* involvement confirmed by molecular analysis. Of note, each of the previously reported cases and our case occurred in pediatric patients, and five out of eight of the cases carried chromosome 21 abnormalities, including constitutional and/or acquired +21 and i(21q).

**Table 1:** Cases of B-ALL bearing a t(2;12)(p11–p13;p12–p13) observed by conventional cytogenetic analysis

Age/Sex	Karyotype	Reference
9/M	47,XY,+21c[25]/47,idem,t(2;12)(p12;p13),t(8;14)(q11.2;q32)[5]	Index case
2/F	46~47,XX,t(2;12)(p13;p13),+del(3)(q12q25),-5,del(6)(q21),-9,+1~2mar[5]/46,XX[12]	[15]
8/M	46,XY,t(2;12)(p13;p12),t(9;10)(p22;q21)[21]	[16]
Pediatric/M	50,XY,+X,t(1;15)(q42;q15),t(2;12)(p11;p13),+8,+10,+14,-20,i(21)(q10),+i(21)(q10)	[17]
Pediatric/F	49,XX,+10,+16,+21,del(1)(q12),t(2;12)(p11;p12),del(7)(p11)	[18]
Pediatric/M	46,XY,t(2;12)(p11;p12)	[18]
2/F	58,XY,+X,t(2;12)(?p13;p13),+4,+6,+8,+10,+11,+14,+17,+18,+21,+21,+mar[5]/46,XY[4]	[19]
Pediatric/M	66,XY,+X,+Y,t(2;12)(p11;p13),+4,+5,+6,+8,+10,+11,+12,+14,+14,+15,+16,+17,+18,+21,+21,+21,+22[6]/46,XY[15]	[20]

*ETV6* is able to promote leukemogenesis via a variety of mechanisms. Most commonly, *ETV6* is involved in translocations with partner genes that encode protein tyrosine kinases or transcription factors, leading to deregulation of signaling pathways essential to hematopoiesis via generation of fusion genes or aberrant activation of proto-oncogenes [3]. Additionally, most evidence supports the notion that *ETV6* functions as a tumor suppressor gene. For example, in cases where one allele of *ETV6* is involved in a translocation, there is often a concomitant deletion of the other, non-rearranged allele [3]. Additionally, decreased or absent *ETV6* expression has been observed in cases that don’t bear a deletion of *ETV6*. Finally, point mutations leading to loss of function of *ETV6* have also been observed [3]. These lines of evidence suggest that the t(2;12)(p12; p13) in our case is likely a translocation involving *ETV6* and an unknown partner gene on the short arm of chromosome 2 leading to aberrant activation of a protein tyrosine kinase pathway or a proto-oncogene encoded by that unknown partner gene. Alternatively, this rearrangement could result in loss of function of *ETV6* without affecting or involving a partner gene, which has also been shown to contribute to leukemogenesis [3].

However, two reported cases – one case of myelodysplastic syndrome and one case of B-ALL – showed amplification of the *ETV6* gene via the generation of homogeneously staining regions (hsr) on the short arm of chromosome 12 [4, 5]. In both of these cases, further molecular analysis confirmed that the homogeneously staining regions consisted primarily of *ETV6* gene material, confirming the amplification, and immunohistochemical analysis confirmed overexpression of *ETV6* compared to case-matched controls [4, 5]. Furthermore, no additional mutations involving *ETV6* that are known or predicted to result in its loss of function were observed in these cases. These two cases provide evidence against the hypothesis that *ETV6* functions exclusively as a tumor suppressor gene and suggests that in certain contexts, it can function as an oncogene via its overexpression, a rare phenomenon that has been observed in other genes such as the Wilms tumor 1 (WT1) gene [11]. Because the t(2;12)(p12; p13) observed in MCL involves overexpression of *CCND2* due to placement of the gene near the regulatory regions of *IGK*, it is possible that in our case, a similar mechanism could result in overexpression of *ETV6* in the context of this translocation due to the placement of *ETV6* near the regulatory regions of *IGK*. Additionally, Lu *et al.* reported the first case of B-ALL bearing a t(12;14)(p13;q32) involving *ETV6* and *IGH@* confirmed by metaphase FISH using probes specific to both genes, showing that translocations involving *ETV6* and immunoglobulin genes can occur in B-cell neoplasias [12]. However, such immunoglobulin translocations can also result in mutation of the partner gene in addition to aberrant expression, which can affect the wild-type function of a tumor suppressor gene [13]. Unfortunately, due to limited sample material, we were not able to conduct further molecular or functional analysis of this case to determine the precise nature of the t(2;12) and the role of *ETV6* in the evolution of this malignancy.

**Fig. 6:**
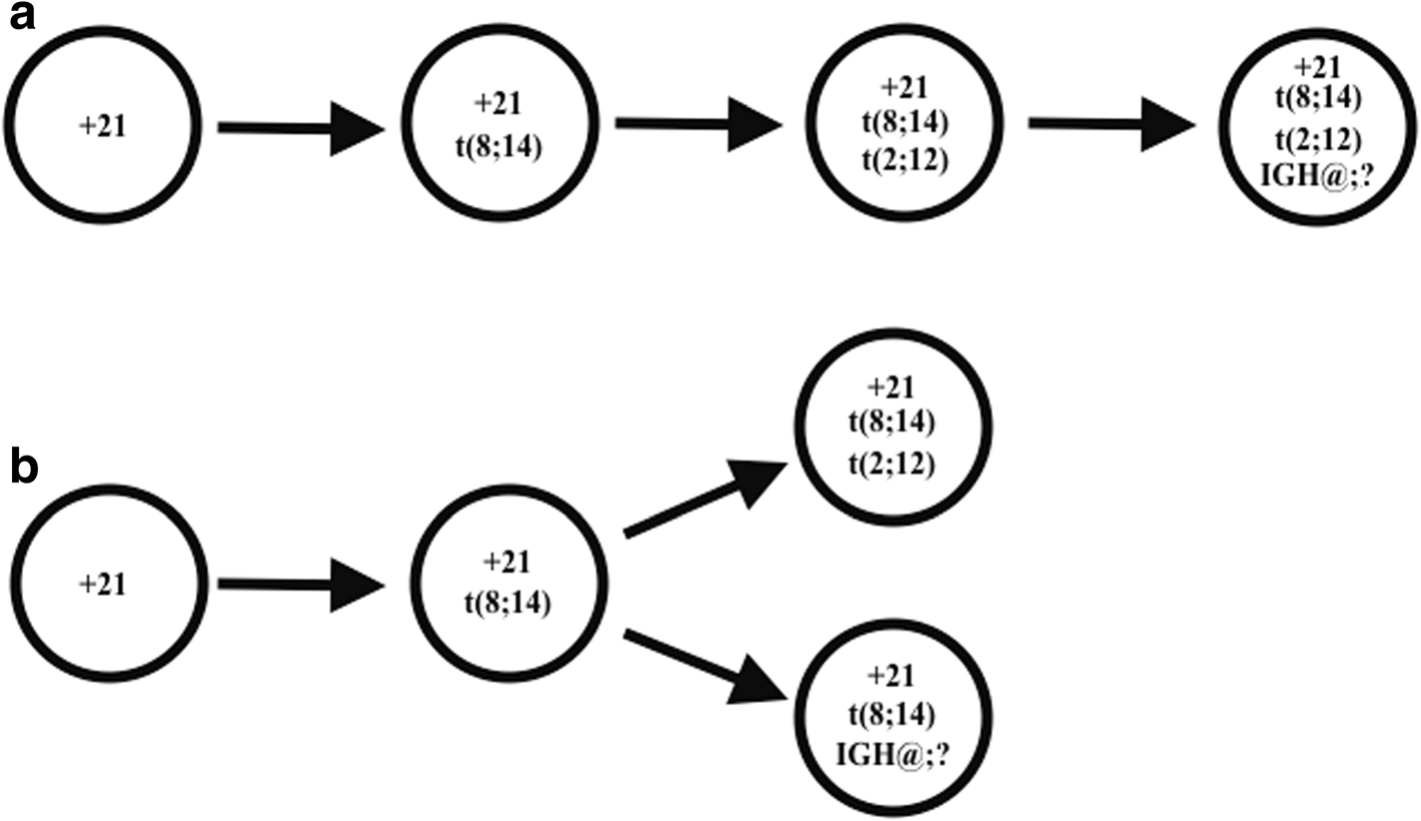
Two most likely mechanisms for the cytogenetic evolution of this malignancy. (**a**) linear cytogenetic evolution (**b**) divergent cytogenetic evolution

The variation of abnormalities of the *IGH@* and *ETV6* genes and the variation in the frequencies of these abnormalities as detected by interphase FISH provides evidence for an underlying cytogenetic evolution in the development of this malignancy. First of all, clones bearing a monoallelic *IGH@* rearrangement, evidenced by a 1F1R1G signal pattern by interphase FISH using an *IGH@* break apart probe, were observed in 89.7 % of interphase nuclei analyzed. Conversely, only 6.7 % of cells showed evidence of biallelic *IGH@* rearrangements (2R2G) using the same probe and 50.7 % of interphase nuclei showed evidence of an *ETV6* rearrangement when analyzed with a dual color, dual fusion translocation probe, suggesting that a monoallelic *IGH@* rearrangement, caused by t(8;14)(q11.2;q32), was the primary cytogenetic abnormality in this malignancy. Because we were not able to detect biallelic 14q32 rearrangements by conventional cytogenetic analysis but were able to observe both a monoallelic t(8;14)(q11.2;q32) and t(2;12)(p12;p13) in the same metaphase spread, the evolution of this malignancy likely happened via one of two mechanisms: (1) acquisition of a primary t(8;14), followed by t(2;12) in the same clone, followed by an additional *IGH@* rearrangement in the same clone or (2) acquisition of a primary t(8;14), followed by t(2;12) in one clone and an additional *IGH@* rearrangement in a different clone (Fig. 6). The former mechanism, depicted in Fig. 6a, would result in a linear cytogenetic evolution such that a clonal population would exist that bears all three abnormalities observed in this case: a t(8;14), a t(2;12), and an additional *IGH@* rearrangement. Conversely, the latter mechanism, depicted in Fig. 6b, would result in a divergent cytogenetic evolution such that one clonal population would bear a t(8;14), a t(2;12), and no additional *IGH@* rearrangement and the other would bear a t(8;14) and an additional *IGH@* rearrangement, but not a t(2;12). It is important to note that because we did not observe biallelic 14q32 rearrangements in any metaphase spreads by conventional cytogenetic analysis or metaphase FISH, the additional *IGH@* rearrangement could be due to an additional t(8;14)(q11.2; q32) or an *IGH@* rearrangement involving a different gene or locus. Due to the relatively low frequency of the clone bearing biallelic *IGH@* rearrangements, it is more likely that this malignancy evolved via the former, linear mechanism, although both possibilities must be considered.

Among all reported cases of t(8;14)(q11.2; q32)-ALL, only one case, reported by Harrison *et al.*, was found to bear a concomitant cytogenetic abnormality involving the 12p13 locus, described as follows: 47, XX, t(8;14)(q11; q32), del(12)(p12p13),+21c. Further FISH studies confirmed both a monoallelic *IGH@* rearrangement and a monoallelic *ETV6* deletion [14]. It is evident that the involvement of cytogenetically detectable abnormalities involving *ETV6* in the evolution of t(8;14)(q11.2; q32)-ALL is an extremely rare occurrence that presents a number of questions regarding molecular mechanisms and clinical implications that remain understudied. We emphasize the importance of utilizing both conventional cytogenetic and molecular genetic tools to elucidate relevant abnormalities and molecular mechanisms in such cases, ultimately to determine the clinical implications of rare cytogenetic abnormalities in pediatric ALL such as those presented in this case. In summary, this case provides insight into a novel translocation involving *ETV6* as well as potentially unique and understudied mechanisms of clonal evolution in pediatric B-ALL that warrant further investigation.
