# Erratum to: A case of B-cell acute lymphoblastic leukemia in a child with Down syndrome bearing a t(2;12)(p12;p13) involving ETV6 and biallelic IGH@ rearrangements

**DOI:** 10.1186/s40364-015-0052-1

**Published:** 2015-11-02

**Authors:** Carlos A. Tirado, David Shabsovich, Yeun Kim, Peter Traum, Sheeja Pullarkat, Michael Kallen, Nagesh Rao

**Affiliations:** Department of Pathology and Laboratory Medicine, David Geffen School of Medicine at UCLA, Los Angeles, CA USA

The original version of this article [1] unfortunately contained mistakes.

The metaphase FISH studies using the Vysis IGH Dual Color, Break Apart Rearrangement Probe revealed 1 fusion signal on the normal chromosome 14 corresponding to an intact IGH@ locus, a red signal corresponding to the 3′ region of the IGH@ locus on the derivative chromosome 14, and a green signal corresponding to the 5′ region of the IGH@ locus on the derivative chromosome 8. This was not described correctly in the results section of the original manuscript.

Additionally, in the discussion, the reference for the case bearing the concomitant t(8;14)(q11.2;q32) and del(12)(p12p13) should be Byatt et al., not Harrison et al.

Finally, the email of the corresponding author (Carlos Tirado) should be ctirado@mednet.ucla.edu.
